# Genome-Wide Identification and Characterization of the GRF Gene Family in *Melastoma dodecandrum*

**DOI:** 10.3390/ijms24021261

**Published:** 2023-01-09

**Authors:** Jie Huang, Gui-Zhen Chen, Sagheer Ahmad, Yang Hao, Jin-Liao Chen, Yu-Zhen Zhou, Si-Ren Lan, Zhong-Jian Liu, Dong-Hui Peng

**Affiliations:** Key Laboratory of National Forestry and Grassland Administration for Orchid Conservation and Utilization at Landscape Architecture and Arts, Fujian Agriculture and Forestry University, Fuzhou 350002, China

**Keywords:** GRF family, genome-wide identification, growth and development, *Melastoma dodecandrum*, flowering

## Abstract

Growth-regulating factor (GRF) is a kind of transcription factor unique to plants, playing an important role in the flowering regulation, growth, and development of plants. *Melastoma dodecandrum* is an important member of Melastomataceae, with ornamental, medicinal, and edible benefits. The identification of the GRF gene family in *M. dodecandrum* can help to improve their character of flavor and continuous flowering. The members of the GRF gene family were identified from the *M. dodecandrum* genome, and their bioinformatics, selective pressure, and expression patterns were analyzed. The results showed that there were 20 GRF genes in *M. dodecandrum*. Phylogenetic analysis showed that the 71 GRF genes from *M. dodecandrum*, *Arabidopsis thaliana*, *Camellia sinensis*, and *Oryza sativa* can be divided into three clades and six subclades. The 20 GRF genes of *M. dodecandrum* were distributed in twelve chromosomes and one contig. Furthermore, the gene structure and motif analysis showed that the intron and motif within each clade were very similar, but there were great differences among different clades. The promoter contained cis-acting elements related to hormone induction, stress, and growth and development. Different transcriptomic expression of MdGRFs indicated that MdGRFs may be involved in regulating the growth and development of *M. dodecandrum*. The results laid a foundation for further study on the function and molecular mechanism of the *M. dodecandrum* GRF gene family.

## 1. Introduction

Growth-regulating factors (GRFs) are transcription factors unique to plants that play a significant role in plant growth and development. They were first discovered in *Oryza sativa* in 2000 [1]. Recently, GRF family members have been studied in some plants, such as *Zea mays* [2], *C. sinensis* [3], *Arabidopsis* [4], *Brassica rapa* [5], *Brassica napus* [6], *Solanu lycopersicum* [7], *Nicotiana tabacum* [8], *Manihot esculenta* [9], *Malus pumila* [10], Cucurbitaceae [11], *Brachypodium distachyon* [12], *Morus alba* [13], Juglandaceae [14], and *Nelumbo nucifera* [15]. The GRF gene family plays an important role in the formation of tissues and organs in various plant biological processes, especially during the early growth period, such as leaf development [16], root growth [17], stem elongation [18], floral organ maturation [19], seed formation [20], and hormone signal transduction [21]. Previous studies have shown that the overexpression of *AtGRF1* and *AtGRF2* can lead to the enlargement of leaves and cotyledons, and that *AtGRF8* is related to flower development in *Arabidopsis* [4]. The GRF gene family in *S. lycopersicum* is widely involved in the growth process, such as in the increased expression of *SlGRF1* during fruit ripening, higher expression of *SlGRF10* in biological tissue, higher expression of *SlGRF4* and *SlGRF8* in flower buds, and the highest expression of *SlGRF6* in root [7]. The expression of *AhGRF5* was higher in the rapid expansion stage of the pod in peanuts [22].

The GRF gene family has specific highly conserved domains, which are mainly located in the N-terminal, QLQ (Glu-Leu-Glu), and WRC (Trp-Arg-Cys) conserved domains [23]. The QLQ domain is highly homologous to the N-terminal of yeast SWI2/SNF2 protein and is an important protein–protein interaction domain. The interaction protein GIF (GRF-interacting factor) of *AtGRF1* was identified via the yeast two-hybrid technique [24]. WRC is a kind of plant-specific motif, which is responsible for the interaction between transcription factors and DNA in the nucleus. It contains a DNA-binding nucleus localization signal region and zinc finger structure (C-X9-C-X10-C-X2-H), which regulates the expression of downstream target genes [2,4,5,8,25]. However, research on the GRF gene family in *M. dodecandrum* has not been reported.

*M. dodecandrum* is a crucial subshrub in the *Melastoma* genus. It is mainly distributed in southern China. It has great ornamental value and is also used as a folk herbal medicine. The plants of *M. dodecandrum* are low and creeping, with thick leaves close to the ground which can form a flat and dense ground cover. Their leaves, flowers, and fruits show variable colors throughout the year. It has high ornamental value and has resistance to shade and trampling. It can be used as an excellent ground cover plant in the popularization and application of landscape architecture [26]. The fruit of *M. dodecandrum* is a kind of juicy berry with a highly nutritive value containing vitamin C, crude protein, cyanin, and so on [27,28]. Studying the GRF family can do a great deal to understand the growth and development pattern of this species and improve its character. Therefore, we report the identification and expression analysis of the GRF gene family in *M. dodecandrum*.

## 2. Results

### 2.1. Identification and Physicochemical Property Analysis of GRF

Our study used the amino acid sequences of *Arabidopsis* and rice GRF genes as a reference. Twenty GRF genes were identified. Based on the analysis of the physicochemical properties of the GRF gene family of *M. dodecandrum*, it was found that the physicochemical properties of each member were different. The length of the GRF gene family of *M. dodecandrum* ranged from 277 to 637 aa (Table 1). The molecular weight was from 29,999.53 to 68,659.67 Da. The isoelectric point of the GRF gene family was from 5.94 to 9.3. The instability index was from 44.32 to 74.3. The grand average of hydropathicity was from −0.859 to −0.475.

### 2.2. Conserved Domain and Gene Structure Analysis of GRF

**Figure 1:**
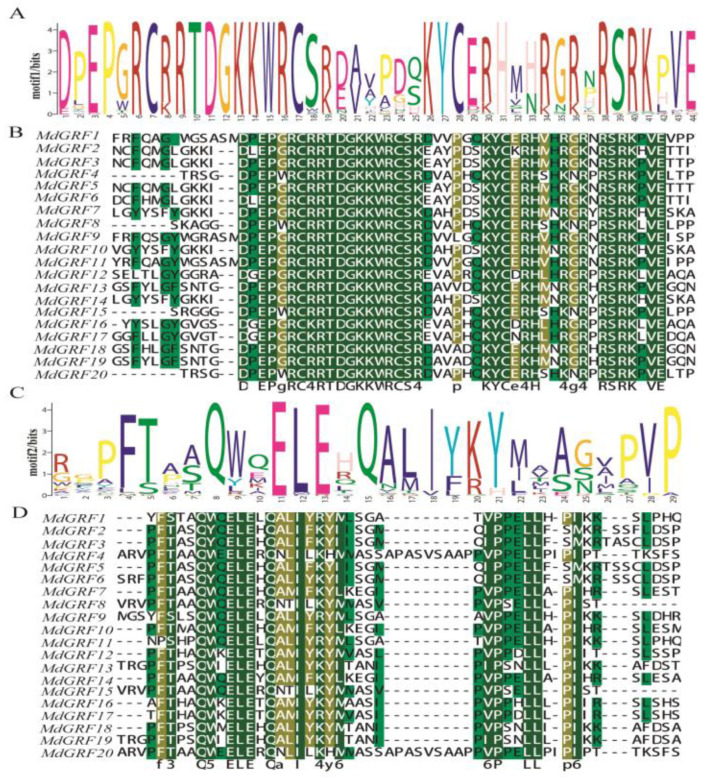
Conserved domains and Seqlogos of GRF gene family in *M. dodecandrum*. (**A**), Seqlogo of WRC (motif 1); (**B**), conserved sequence alignment of WRC; (**C**), Seqlogo of QLQ (motif 2); (**D**), conserved sequence alignment of QLQ.

**Figure 2:**
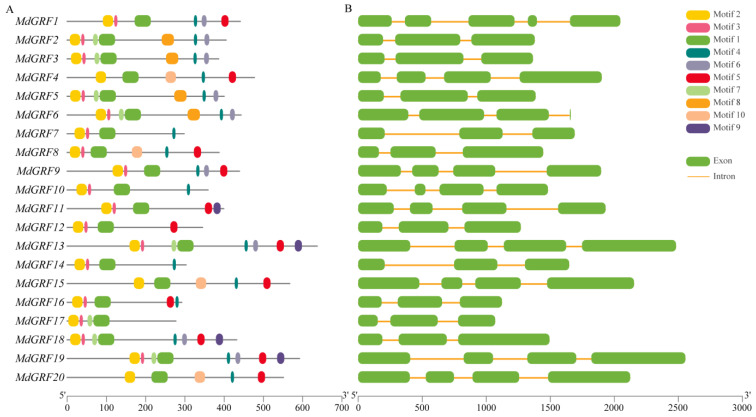
Motifs and gene structure of GRF gene family in *M. dodecandrum*. (**A**), motifs of MdGRF; (**B**), exon and intron of MdGRF.

The analysis of the conserved domain showed that all of the GRF genes of *M. dodecandrum* had two completely conserved domains: WRC (Figure 1A,B) and QLQ (Figure 1C,D). Ten conserved motifs in the GRF gene family were identified using MEME online tools (Figure 2A). The results showed that all genes of the GRF family had two typical motifs, motif 1 (WRC) and motif 2 (QLQ), indicating that these two motifs were relatively conservative among the GRF gene family (Figure 1 and Figure 2A). With the exception of *MdGRF4* and *MdGRF20*, all members of the GRF gene family had conserved motif 3. Except for *MdGRF17*, *MdGRF12*, and *MdGRF11*, the other 17 GRF genes contained motif 4. *MdGRF10* and *MdGRF7* contained the least conservative motifs, indicating that the GRF gene family of *M. dodecandrum* may have a complex evolutionary process. 

The gene structure of the GRF gene family showed diversity (Figure 2B). The number of GRF genes’ exons of *M. dodecandrum* ranged from 3–5. Moreover, seven genes (*MdGRF2*, *MdGRF3*, *MdGRF6*, *MdGRF5*, *MdGRF14*, *MdGRF17*, and *MdGRF7*) contained two introns, *MdGRF1* contained four introns, and the other GRF genes contained three introns. 

### 2.3. Cis-acting element prediction of GRF gene family

**Figure 3:**
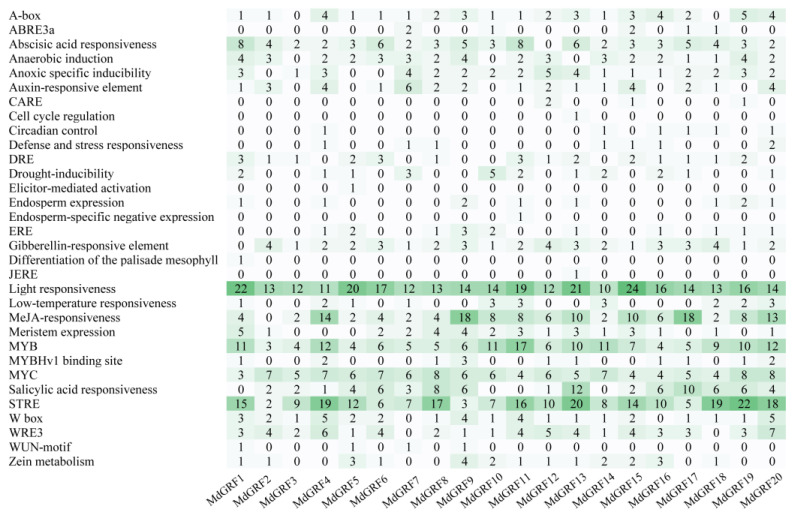
Types and numbers of cis-acting elements in promoters of *M. dodecandrum* GRF genes. Note: The darker the color, the greater the value.

The results of the GRF gene family of promoter acting element prediction of the upstream 2000 bp sequence showed that there were a variety of environmental and stress response elements in the GRF gene family of *M. dodecandrum* (Figure 3). All MdGRF genes contained light response elements (307), STREs (239), MYB biding sites (158), and MYC biding sites (116); 95% of MdGRF genes contained methyl jasmonate response element (141), abscisic acid responsiveness element (74), and gibberellin-responsive element (44); 90% of MdGRF genes contained WRE3 element (58), A-box (40), and W-box (38); 85% of MdGRF genes contained anaerobic induction element (43) and anoxic specific inducibility element (40); 80% of MdGRF genes had salicylic acid responsiveness elements (79); 75% of MdGRF genes had auxin-responsive element (35) and meristem expression element (34); 70% of MdGRFs contained DRE element (24); 65% MdGRFs had zein metabolism element (23); 55% MdGRF genes had drought inducibility element (21); 50% of MdGRFs contained low-temperature responsiveness element (21), ERE element (14), MYBHv1 binding site element (14), and endosperm expression element (10); 35% of MdGRF genes had defense and stress responsiveness element (8); 30% of MdGRF genes had circadian control element (6); 25% of MdGRF genes had ABRE3a element (7); 20% of MdGRFs had WUN-motif (4); and 10% of MdGRFs had CARE element (4). Furthermore, only 5% MdGRF genes had cell cycle regulation element (1), elicitor-mediated activation element (1), endosperm-specific negative expression element (1), differentiation of the palisade mesophyll element (1), and JERE element (1).

### 2.4. Phylogeny and Evolution Analysis of GRF Gene Family

**Figure 4:**
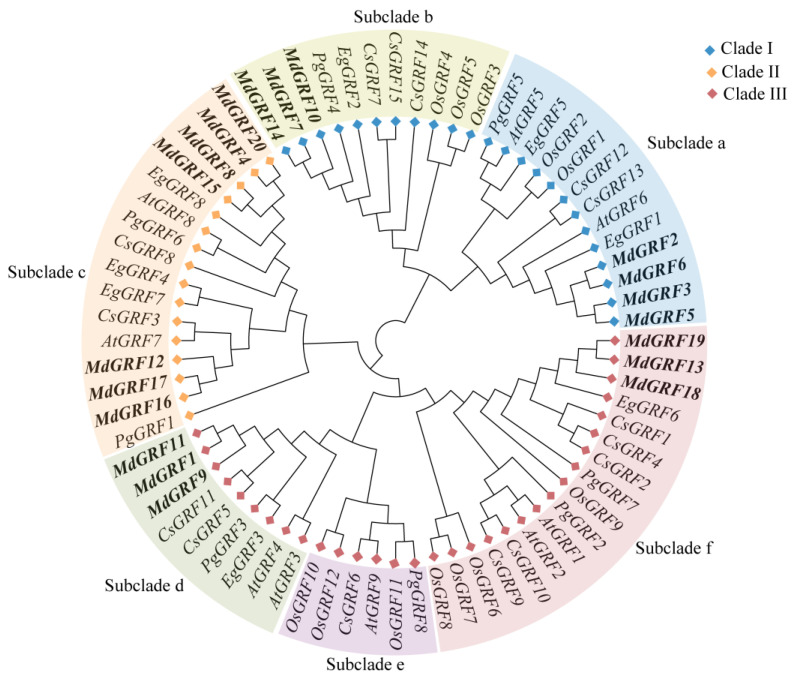
Phylogeny analysis among *M. dodecandrum*, *C. sinensis*, *A. thaliana*, *E. grandis*, *P. granatum*, and *O. sativa*. Note: gene name initial of ‘At’ are genes of *A. thaliana*, ‘Md’ are genes of *M. dodecandrum*, ‘Os’ are genes of *O. sativa*, ‘Cs’ are genes of *C. sinensis*, ‘Eg’ are genes of *E. grandis*, and ‘Pg’ are genes of *P. granatum*.

The phylogenetic tree was constructed with GRF members of *M. dodecandrum* (20), *C. sinensis* (15), *A. thaliana* (9), *E. grandis* (8), *P. granatum* (8), and *O. sativa* (9) [1]. The result showed that these 71 GRF genes could be divided into three clades (Clades I-III) and six subclades (subclades a–f) (Figure 4). Clade I contained two subclades (a–b), Clade II only had one subclade (c), and Clade II contained three subclades (d–f). In subclade a, there were four MdGRFs, two EgGRFs, two AtGRFs, two OsGRFs, two CsGRFs, and one PgGRF. There were three MdGRFs, one EgGRF, three OsGRFs, three CsGRFs, one PgGRF, and no AtGRF found in subclade b. In subclade c, there were seven MdGRFs, three EgGRFs, three OsGRFs, two CsGRFs, and two PgGRFs, while there was no OsGRF in this subclade. Subclade d had three MdGRFs, one EgGRF, two CsGRFs, one PgGRF, and two AtGRFs, while there was no OsGRF in this subclade. Subclade e contained three OsGRFs, one AtGRF, one PgGRF, and one CsGRF, but no MdGRF or EgGRF were found in this subclade. There were three genes of MdGRFs, one EgGRF, five CsGRFs, four OsGRFs, three CsGRFs, two PgGRFs, and two AtGRFs in the subclade f. 

### 2.5. Gene Chromosome Mapping and Collinearity Analysis

**Figure 5:**
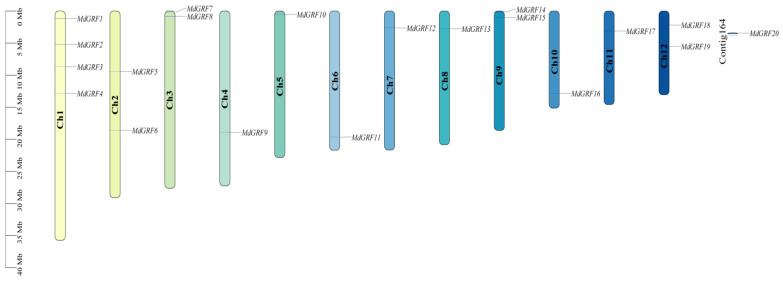
Chromosome localization of GRF gene family of *M. dodecandrum*.

Chromosome mapping showed that 20 GRF genes were unevenly distributed on 12 chromosomes of *M. dodecandrum* (Figure 5). There were four genes (*MdGRF1*, *MdGRF2*, *MdGRF3*, and *MdGRF4*) in Chromosome 1. Chromosome 2, Chromosome 3, Chromosome 9, and Chromosome 12 all contained two genes. Only one gene was present on Chromosome 4, Chromosome 5, Chromosome 6, Chromosome 7, Chromosome 8, Chromosome 10, and Chromosome 11. The remaining one gene was located in the contig 164.

**Figure 6:**
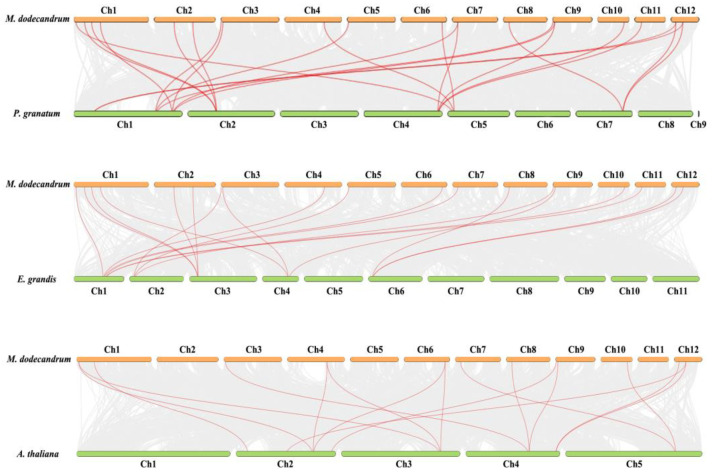
Synteny analysis of GRF gene family between *M. dodecandrum* and *A. thaliana*, *E. grandis* and *P. granatum*. Note: gray lines represent colinear gene pairs between *M. dodecandrum* and other species, and red lines represent colinear GRF gene pairs.

In order to understand the evolutionary mechanism of the GRF gene family of *M. dodecandrum*, we analyzed the collinear relationship between *M. dodecandrum* and *E. grandis*, and *P. granatum* (Figure 6). The results show that 22 pairs of collinearity genes of GRF were between *M. dodecandrum* and *P. granatum*, followed by *M. dodecandrum* and *E. grandis* (19 pairs), and the least was *M. dodecandrum* and *A. thaliana* (15 pairs). 

According to the result of evolution analysis, the values of Ka, Ks, and Ka/Ks were obtained (Table 2). Nineteen gene pairs were identified using Tbtools. The value of Ka/Ks of each pair ranged from 0.155721552 to 0.44840491 (Ka/Ks < 1). This result indicated that all of them had undergone strong purifying selection. 

### 2.6. Expression Pattern Analysis of GRF Gene Family 

**Figure 7:**
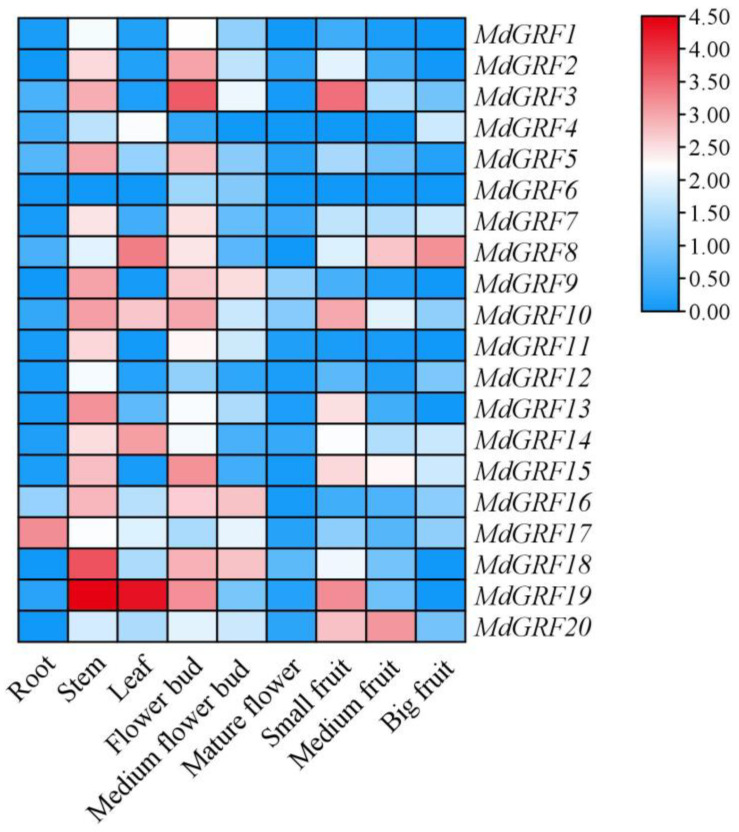
Heatmap of GRF in *M. dodecandrum*. Note: different colors represent different FPKM values; the color gradient from blue to red represents increasing expression level.

Based on the transcriptome data of *M. dodecandrum*, the tissue expression pattern of the GRF gene family was analyzed, and nine samples including root, stem, leaf, flower bud, medium flower bud, mature flower, small fruit, medium fruit, and big fruit were selected for prediction. The result showed that the MdGRF genes were expressed differently in the nine different samples. The GRF genes were expressed in root, stem, leaf, flower bud, medium flower bud, mature flower, small fruit, medium fruit, and big fruit, especially in the stem, leaf, flower bud, medium flower bud, and small fruit (Figure 7). *MdGRF19* was highly expressed in the stem and leaf, and showed normal expression in the flower bud and small fruit and low expression in the other samples. *MdGRF17* was highly expressed in the root, while it showed a medium or low expression in the other samples. *MdGRF8* showed significant expression in the leaf and big fruit, while medium or low expression was observed in the other samples. A high expression of *MdGRF18* was observed in the stem, flower bud, and medium flower bud, while a medium or low expression was seen in the other samples. Nearly the same pattern was observed for *MdGRF1*, *MdGRF2*, *MdGRF3*, *MdGRF5*, *MdGRF7*, and *MdGRF11*, which had high expression in flower bud and stem, and were nearly not expressed in root, leaf, medium flower bud, mature flower, small fruit, medium fruit, and big fruit. However, the expression of *MdGRF6* was very low in the nine different samples. It is speculated that the difference in its expression may be related to the mechanism of plant growth and the development being regulated by the GRF gene family.

**Figure 8:**
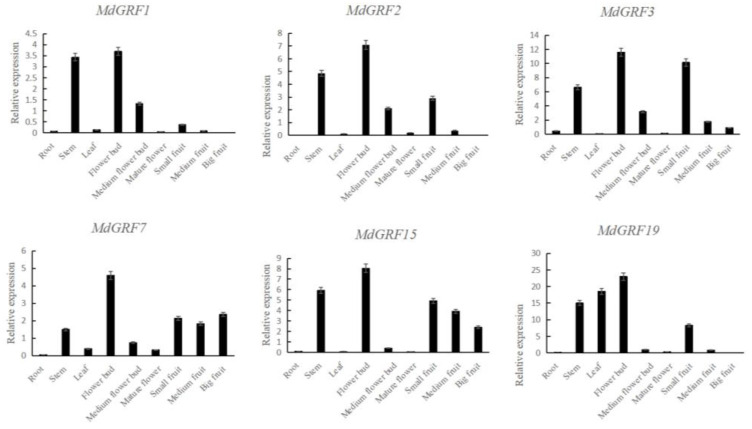
Relative expression for MdGRFs in different organs. Note: abscissa represents different organs; ordinate represents value of relative expression.

RT-qPCR was performed to assess the accuracy of transcriptome sequencing of all nine samples in *MdGRF1*, *MdGRF2*, *MdGRF3*, *MdGRF7*, *MdGRF15,* and *MdGRF19*. The result of RT-qPCR showed high expression in the flower bud, stem, and small fruit (Figure 8). Three genes (*MdGRF1*, *MdGRF2*, and *MdGRF3*) showed nearly the same between the result of RT-qPCR and transcriptome data, and *MdGRF7* and *MdGRF15* were nearly the same. However, with the gene of *MdGRF19*, it was expressed the highest in the stem according to the transcriptome data, and was medium expressed in the flower. However, according to the result of RT-qPCR, *MdGRF19* was expressed the highest in the flower bud. These differences may be caused by the imperfect correlation between sequencing and RT-qPCR samples. 

### 2.7. Subcellular Localization Analysis of MdGRF19

**Figure 9:**
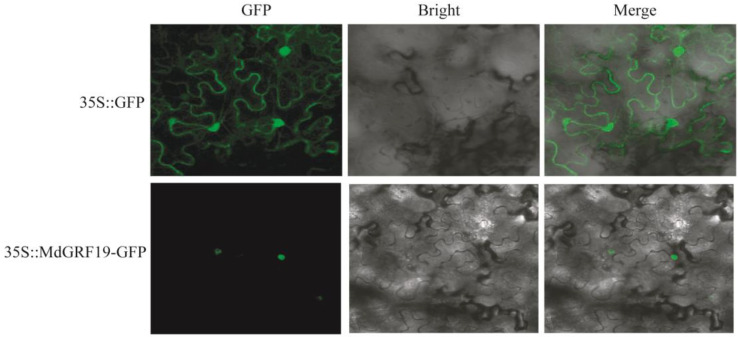
Subcellular localizations of *MdGRF19* in tobacco leaf. Scale bars = 10 µm. 35S::GFP was used as the empty control.

In this study, all MdGRF proteins were predicted to target the nucleus (Table 1). To identify the subcellular localization of MdGRF proteins, we random cloned the *MdGRF19*. One fusion vector was constructed and then transformed into tobacco leaf. The results show that 35S::MdGRF19-GFP was detected as being localized to the nuclear and membrane (Figure 9), which was consistent with the prediction result (Table 1). Moreover, the GFP of the empty protein (35S::GFP) was used as a control group, which was localized to nuclear and cell membranes.

## 3. Discussion

Plant GRF transcription factors play an important regulatory role in plant growth [29]. Our study identified 20 GRF genes of *M. dodecandrum.* Remarkably, the number of GRF genes of *M. dodecandrum* was far more than *A. thaliana* (9), citrus (9), *E. grandis* (8), *P. granatum* (8), and *O. sativa* (9) [1,24], revealing that the GRF genes of *M. dodecandrum* might have undergone large-scale duplication events during evolution. An analysis of the physicochemical properties of GRF in *M. dodecandrum* show that their GRAVY was less than zero, indicating that the MdGRFs belong to hydrophilic protein. Moreover, the instability index of MdGRFs was more than 40, showing that the structures of MdGRF proteins were unstable. 

The previous study indicated that the structure of exon-intron plays an important role in understanding the relationships between evolutionary and functional differentiation [30,31,32]. Furthermore, exon or intron gain/loss events create the gene structure divergence and functional differentiation [33,34]. The structural analysis of MdGRF genes showed that there was little difference in the number and distribution of exons (3–5) and introns (2–4), which indicated that the GRF gene family of *M. dodecandrum* was highly conserved in the process of evolution. There are two to three introns in *A. thaliana* [4]; one to three introns are found in *Brachypodium distachyon* [12], while two to four introns are found in *O. sativa* [1], soybean [35], and wheat [36], which is consistent with *M. dodecandrum*. Moreover, different plants contain different numbers of exons; for example, *AtGRF07* contains five exons, while *MdGRF2* only has two exons. The results showed the diversity in the GRF genes of different plants. Two conserved domains (QLQ/WRC) at the N-terminal were predicted in the MdGRF protein sequences, which was consistent with the results of the study that found that QLQ plays a role in the protein–protein interaction and WRC effectively regulates the transcription of DNA binding c. These results indicated that MdGRF proteins were evolutionarily conserved in plants. 

The result of phylogenic analysis showed that 71 GRF genes could be divided into three clades and six subclades. However, the GRF gene family of *M. dodecandrum* and *A. thaliana* was only divided into five subclades, and no gene was located in the subclades e and b, respectively. Moreover, OsGRF only had four subclades, and lost subclades c and d. We speculated that this phenomenon may be caused by a special gene expansion event (lost or obtained) during the evolutionary process [3]. The phylogenic analysis of GRF gene families in *M. dodecandrum*, *A. thaliana*, *C. sinensis*, *E. grandis*, *P. granatum*, and *O. sativa* showed that *M. dodecandrum* had higher homology with *P. granatum* and *E. grandis*, but a distant genetic relationship with *O. sativa* and *C. sinensis*, which revealed that some ancestor GRF genes existed before the divergence of *M. dodecandrum*, *P. granatum*, and *E. grandis* during evolution. Additionally, these differences also indicated that there may be structural and functional differentiation of the GRF family in dicot and monocot plants, which needs further study. Strong synteny was detected in the *M. dodecandrum* and *P. granatum*, followed by *M. dodecandrum* and *E. grandis*, and the last was the *M. dodecandrum* and *Arabidopsis* genomes. The Ka/Ks of the 19 gene pairs indicated that strong purifying selection may be largely responsible for maintaining the functions of GRF proteins, the same with *A. thaliana* and *O. sativa* [1,24].

GRFs are a key regulator of plant growth and development [37,38]. Previous study has shown that GRFs endow proliferation and meristem potential in the process of organogenesis [39]. Study has also shown that the GRF gene can regulate leaf area by controlling cell proliferation, thus enhancing the adaptation to stress conditions such as drought and high temperature [40]. The cis-acting element analysis showed that MdGRFs members could be transcribed and expressed under different stresses such as low temperature, drought, and anaerobic stress, and regulated under gibberellin and auxin stimulation signals. Some genes were involved in the growth regulation of meristem, embryo, and seed growth. The previous studies show that the expression level of the GRF gene family in growing zones is significantly higher than that in mature tissues [3,4,24], such as the OsGRFs in rice being strongly expressed in buds, immature leaves, and flower buds [25]. The expression of the GRF gene family is highest in the tender organ of *Camellia sinensis*, followed by the stems and immature leaves, and is hardly expressed in the root and mature flower [3]. In *M. dodecandrum*, the expression analysis according to RT-qPCR and transcriptome data of the GRF gene family showed that most MdGRFs were highly expressed in stem, flower bud, and small fruit, while MdGRFs were nearly not expressed in mature flower, root, and mature fruit, which is similar to previous studies on other species [3,25]. The high expression in the tender organ of the GRF gene family in *M. dodecandrum* indicated that MdGRFs may play an important role in regulating the development of plants.

## 4. Materials and Methods

### 4.1. Data Sources

Tender leaf, tender root, tender stem, small fruit, medium fruit, big fruit, flower bud, medium flower bud, and mature flower of wild *M. dodecandrum* were sampled for qPCR. The genome, transcriptome data, and GFF file used in this study follow Hao et al. [41]. Hisat [42] and Stringtie2 [43] were used to align and assemble the transcriptome data of *M. dodecandrum*.

### 4.2. Identification and Physicochemical Property Analysis of GRF Gene Family in M. dodecandrum

*A. thaliana*, *E. grandis*, *P. granatum*, and *O. sativa* GRF gene families were used as reference. GRF proteins of *A. thaliana* and *O. sativa* were downloaded from NCBI (https://www.ncbi.nlm.nih.gov/orffinder/, accessed on 17 May 2022). The Blastp alignment of GRF gene family of *M. dodecandrum* was carried out using TBtools [44], and the E value was less than le-5. The obtained MdGRF protein sequences were listed on CDD (https://www.ncbi.nlm.nih.gov/cdd, accessed on 8 May 2022) to detect their domains, and the protein sequences without QLQ or WRC domain were removed. In addition, two pfam seed models WRC (PF08879) and QLQ (PF08880) were obtained from the online database (http://pfam.xfam.org/, accessed on 8 May 2022) and were used for building a hidden Markov model (HMM) file using HMMER3 software with default parameters. The HMM search program was performed to search for GRF genes from hmm file generated in the previous step. We compared the results of HMM and BLASTP, and removed the repeat genes.

The MdGRF protein sequences were run on the ExPasy website (http://au.expasy.org/tool.html, accessed on 5 May 2022) and analyzed using the Compute pI/MW tool, and a variety of physicochemical properties of GRF protein were obtained, including amino acid length, molecular weight, isoelectric point, and so on [45]. Then, the CELLO v2.5 software (http://cello.life.nctu.edu.tw/, accessed on 17 May 2022) was used to predict the subcellular localization of MdGRF gene family.

### 4.3. Conserved Domain and Gene Structure Analysis of GRF

The gene structure of GRF family of *M. dodecandrum* was analyzed, and the online NCBI Conserve Domain (https://www.ncbi.nlm.nih.gov/cdd/, accessed on 17 May 2022) and MEME (https://meme-suite.org/meme/, accessed on 17 May 2022) were used to predict the conserved domain and motif of GRF gene family [46]. The results were visualized using TBtools (version 1.100, China).

### 4.4. Phylogeny Analysis of GRF Gene Family

Mega7 (version 7.0, America) was used to align the protein sequences of GRF genes and the phylogenetic tree (maximum likelihood) of six species (*M. dodecandrum*, *C. sinensis*, *A. thaliana*, *E. grandis*, *P. granatum*, and *O. sativa*) under a GTRGAMMA substitution model with 1000 bootstraps was constructed [47]. FigTree v1.4.3 was used to edit the tree. Lastly, adobe illustrator was used to beautify the tree.

### 4.5. Chromosome Mapping, Collinearity Analysis

According to the gene location information of GFF annotation file of *M. dodecandrum* genome, the location of GRF genes was mapped using TBtools. We also used TBtools to analyze the collinear relationship between *M. dodecandrum* and *A. thaliana*, *E. grandis*, and *P. granatum*. During evolution, genes may face various selection pressures, positive selection, neutral selection, and purifying selection, for example. This is important for studying the evolution of genes to understand the selection pressure of genes [48]. To understand the evolution of MdGRFs, Ka, Ks, and Ka/Ks were calculated. The simple Ka/Ks calculator in Tbtools (version 1.100, China) was used to analyze it.

### 4.6. Cis-Acting Element Prediction of GRF Gene Family

Tbtools (version 1.100, China) was used to extract the 2000 bp upstream sequences of GRF of *M. dodecandrum* from genomic data. The sequences were uploaded to the online website PlantCART (http://bioinformatics.psb.ugent.be/webtools/plantcare/html/, accessed on 3 May 2022) to predict the Cis-acting elements of GRF of *M. dodecandrum* [49]. The results of PlantCART were used to draw a picture of cis-acting element prediction.

### 4.7. Transcriptome Data and RT-qPCR Analysis of GRF Gene Family

In order to research the possible effect of GRF genes in different organs of *M. dodecandrum*, we analyzed the expression patterns of 20 GRF genes in the leaf, root, stem, small fruit, medium fruit, big fruit, flower bud, medium flower bud, and mature flower. From the transcriptome analysis, we calculated the FPKM of all nine samples, and used TBtools (version 1.100, China) to draw a heatmap.

Six genes (*MdGRF1*, *MdGRF2*, *MdGRF3*, *MdGRF7*, *MdGRF15,* and *MdGRF19*) in four subfamilies were used for RT-qPCR. Total RNA of all nine samples were extracted using the TIANGEN DP441 Reagent (TIANGEN, Beijing, China). Roche detection system (Roche, Switzerland) with SYBR green assays was used for RT-qPCR analysis. Primers and reference gene information for RT-qPCR are listed in Table S1. All experiments were performed in three biological repeats. The relative expression of MdGRFs were calculated using the 2−ΔΔCT method.

### 4.8. Subcellular Localization Analysis

The *MdGRF19* was used further to the subcellular localization analysis. The *MdGRF19* coding sequence (CDS) without stop codon was cloned into pMDC202 vector, which contained a 35s-driven green fluorescent protein (GFP) promoter. The In-Fusion cloning kit named ClonExpress^®^ Ultra One Step Cloning Kit, which is produced by Vazyme, was used to clone. *KpnI* and *XbaI* were the choices utilized as the restriction sites of pMDC202 vector. Then, we transformed the 35S::MdGRF19-GFP into tobacco leaf, and the vector without the gene was used as a control. After 8 h of dark culture, the transformed tobacco was cultured normally (dark for eight hours at 22 °C, light for sixteen hours at 24 °C). After 48 h, the LSM710 confocal laser scanning microscope (CarlZeiss, Jena, Germany) was used to observe the GFP fluorescence signals. The primers of *MdGRF19* used in this study were as follows: forwards: ttggagaggacctcgactctagaATGAGCAGCAGTGGGATGAGCAGAT; reverse: tttttctaccggtaccGATATAATGGAAAAATGAGAAAC.

## 5. Conclusions

This study identified the GRF gene family of *M. dodecandrum* and comparative analysis was performed with *A. thaliana*, *E. grandis*, and *P. granatum*. We studied physicochemical properties, conserved domains, gene structures, phylogeny, cis-acting element prediction chromosome mapping, and collinearity analysis of GRF of *M. dodecandrum*. Then, according to the transcriptome data, we speculated that the GRF gene family of *M. dodecandrum* may play an important role in the development and growth of *M. dodecandrum*, especially in the tender organ. The result of RT-qPCR also supports it. The findings of this study thus provide potential research directions to reveal the role of GRF TFs in the regulation of important ornamental traits of plants.

## Figures and Tables

**Table 1:** Physicochemical properties of GRF gene family proteins in *M. dodecandrum*.

Gene	Gene ID	Number of Amino Acids	Molecular Weight (Average)	PI	Instability Index	Grand Average of Hydropathicity (GRAVY)	Subcellular Localization
*MdGRF1*	DR000066	441	48,554.34	7.34	54.19	−0.693	Nucleus
*MdGRF2*	DR000875	405	45,832.01	9.19	61.19	−0.76	Nucleus
*MdGRF3*	DR001546	386	43,184.82	8.69	57.13	−0.816	Nucleus
*MdGRF4*	DR022817	399	44,121.32	9.3	57.08	−0.809	Nucleus
*MdGRF5*	DR009489	292	31,752.63	7.2	44.85	−0.475	Nucleus
*MdGRF6*	DR005587	592	63,117.05	8.12	49.33	−0.518	Nucleus
*MdGRF7*	DR017530	387	42,203.03	6.85	61.5	−0.674	Nucleus
*MdGRF8*	DR016167	277	29,999.53	7.72	44.32	−0.569	Nucleus
*MdGRF9*	DR028300	477	51,389.04	6.62	56.85	−0.623	Nucleus
*MdGRF10*	DR013209	567	60,022.76	6.44	67.12	−0.796	Nucleus
*MdGRF11*	DR022630	432	46,814.3	8.02	45.27	−0.542	Nucleus
*MdGRF12*	DR017665	298	33,057.79	8.19	53.3	−0.652	Nucleus
*MdGRF13*	DR030091	439	48,810.12	8.79	69.76	−0.859	Nucleus
*MdGRF14*	DR012552	400	44,738.51	8.97	60.93	−0.839	Nucleus
*MdGRF15*	DR013042	303	33,526.3	8.89	53.52	−0.706	Nucleus
*MdGRF16*	DR006760	443	49,686.1	6.99	74.3	−0.773	Nucleus
*MdGRF17*	DR016005	359	39,646.59	8.67	54.35	−0.585	Nucleus
*MdGRF18*	DR022043	345	36,736.86	8.8	49.51	−0.481	Nucleus
*MdGRF19*	DR034227	637	68,659.67	8.02	54.68	−0.486	Nucleus
*MdGRF20*	DR035468	551	59,247.32	5.94	59.39	−0.662	Nucleus

**Table 2:** Selective pressure analysis of MdGRFs.

Sequence 1	Sequence 2	Ka	Ks	Ka/Ks
*MdGRF1*	*MdGRF11*	0.173493307	0.398825333	0.43501075
*MdGRF2*	*MdGRF5*	0.132485395	0.850783938	0.155721552
*MdGRF2*	*MdGRF6*	0.063855643	0.280841154	0.227372814
*MdGRF3*	*MdGRF5*	0.077035229	0.21431482	0.359448912
*MdGRF3*	*MdGRF6*	0.151447669	0.797959229	0.189793743
*MdGRF6*	*MdGRF5*	0.141888261	0.907851387	0.156290185
*MdGRF8*	*MdGRF4*	0.215952273	0.982105616	0.219887015
*MdGRF10*	*MdGRF7*	0.139852066	0.560101487	0.249690582
*MdGRF11*	*MdGRF9*	0.350866443	0.976254354	0.359400643
*MdGRF14*	*MdGRF10*	0.143791642	0.5827495	0.246746916
*MdGRF14*	*MdGRF7*	0.053995183	0.141992753	0.380267179
*MdGRF15*	*MdGRF4*	0.227932514	0.860771403	0.264800287
*MdGRF15*	*MdGRF8*	0.047974242	0.208451325	0.230146014
*MdGRF16*	*MdGRF12*	0.258405456	0.941249704	0.274534435
*MdGRF16*	*MdGRF17*	0.1223847	0.272933451	0.44840491
*MdGRF17*	*MdGRF12*	0.325439183	0.969576227	0.335650951
*MdGRF18*	*MdGRF13*	0.192896922	0.819618927	0.235349521
*MdGRF19*	*MdGRF13*	0.061481126	0.201014717	0.305853855
*MdGRF19*	*MdGRF18*	0.181514036	0.76674553	0.236733087

## Data Availability

Not applicable.

## Supplementary Materials

The following supporting information can be downloaded at: https://www.mdpi.com/article/10.3390/ijms24021261/s1.
