# Supplementary material for: Genome-Wide Identification and Characterization of the GRF Gene Family in Melastoma dodecandrum
Source: Int J Mol Sci. 2023 Jan 9;24(2):1261. doi: 10.3390/ijms24021261 (PMC9863823; doi:10.3390/ijms24021261)
Supplement: Supplementary file 1 [file ijms-24-01261-s001.zip › Table S1.pdf]

Table S1. The primers for RT-qPCR, the *MdACT* was the reference gene.

Gene name	Primer	Sequence
MdGRF1	Forward	AGTCCTTCGCCACTTCTTCG
	Reverse	TTTCTGGCGGTCGACATTGA
MdGRF2	Forward	TGCATCCCTTTCCATCTCGT
	Reverse	GCACTGGGATAAGCATTGCC
MdGRF3	Forward	AGCCTGTGGAAACAACTCCG
	Reverse	GCATTGCAGGCTGATGAGGA
MdGRF7	Forward	TCGACCACAAAGTCGGATGT
	Reverse	GAGGGCACTTCCAACACCTA
MdGRF15	Forward	GAGCGTCACTCCCACAAGAA
	Reverse	AGCAGGGTTGAGGAGGTAGT
MdGRF19	Forward	AAAGCCTGTGGAAGGCCAAA
	Reverse	AGCAGAGGAAACTGCAGACA
MdACT	Forward	TGCCCTTGACTATGAACAGG
	Reverse	GAATCTCTCAGCACCAATCG
